# Effects of heat stress on physiological, endocrine, and reproductive functions in swine: review

**DOI:** 10.5713/ab.250866

**Published:** 2026-07-27

**Authors:** Jae-Hyeok Lee, Se-Na Jo, Chaiwat Arjin, Jin-Su Kim, Jae-Cheol Jang

**Affiliations:** 1 Division of Animal Science and Institute of Agricultural and Life Science, Gyeongsang National University, Jinju, Korea; 2 Livestock Innovative and Biocirculation Laboratory, Department of Animal and Aquatic Sciences, Faculty of Agriculture, Chiang Mai University, Chiang Mai, Thailand; 3 Department of Animal Industry Convergence, Kangwon National University, Chuncheon, Korea

**Keywords:** Gut Microbiota, Heat Stress, Metabolic Reprogramming, Swine, Temperature–Humidity Index

## Abstract

Heat stress (HS) induced by climate change significantly impacts the productive and reproductive performance of swine. Using temperature-humidity index for quantifying heat load as a framework, this review integrates HS effects across the endocrine and metabolic axis, the gut barrier microbiota, immune axis, and the reproductive axis. HS, within the context of hypothalamic, pituitary, adrenal (HPA) sympathetic–adrenomedullary activation and interactions among insulin, catecholamine, and cortisol crosstalk, results in relative hyperinsulinemia. This condition, via the mediation of pyruvate dehydrogenase kinase 4, inhibits pyruvate dehydrogenase and influences the lactate dehydrogenase/hypoxia-inducible factor-1α (HIF-1α) axis, leading to a redistribution of substrate utilization towards glucose and constraining lipid mobilization. Short chain fatty acids (SCFA) depletion, β-diversity, and centered dysbiosis weaken HIF-1α-dependent mucosal barrier defenses within the gut immune axis. This, along with increased lipopolysaccharide translocation, hyperactivates TLR4/NF-κB signaling, the NLR family pyrin domain containing 3 inflammasome, and Th17/IL-17 pathways. Fecal microbiota transplantation evidence supports a causal relationship between dysbiosis and pathogenesis. Dysregulated crosstalk within the hypothalamic–pituitary–gonadal–HPA axis adversely affects both follicular and luteal function, as well as placental nutrient transport. Intrauterine HS can reprogram F1 offspring, resulting in lasting effects on metabolism, immunity, and reproductive capabilities. In conclusion, HS functions as a systems-level stressor that perturbs multiple physiological axes. Advancement in this field necessitates the development of standardized HS models, the application of multi-omics approaches and multigenerational follow-up studies, and the integration of environmental, nutritional, and management strategies. Shifts in beta diversity the gut microbiota and SCFA depletion emerged as core pathophysiologic mediators. These findings support for their potential use as early biomarkers and intervention targets, underscoring the need for targeted investigation.

## INTRODUCTION

As climate change accelerates and extreme weather becomes more frequent, adapting livestock systems is now essential. Notably, the frequency of summer heat extremes has continued to rise according to the Copernicus ERA5 reanalysis [1], temperature anomalies in summer 2024 relative to the past 30 year mean were +0.67°C in June, +0.68°C in July, and +0.71°C in August. This warming signal is not confined to a single region: over the most recent five year period, the area weighted mean summer temperature increased by +0.76°C in Asia, +0.81°C in Africa, +0.54°C in Europe, +0.25°C in North America, +0.71°C in South America, and +0.41°C in Oceania [1]. Consistent with global trends, Asia also warmed in 2024, with heatwave days increasing by 11.7 days and mean summer temperature increasing by 0.76°C relative to the recent five-year mean across four Asian countries, namely South Korea, China, Japan, and India (Figure 1) [2–5]. These patterns indicate an intensification of summer heat events; sustained exposure to high ambient temperatures depresses livestock productivity, reduces feed intake (FI), and compromises immune competence [6].

Compared with other livestock species, pigs are more susceptible to heat stress (HS) because their thermoregulatory capacity is limited by morphological traits, including a thick subcutaneous fat layer and poorly developed sweat glands, and because decades of selection for high productivity and rapid growth have left them relatively disadvantaged under HS [7]. Accordingly, the recent climate-driven increase in summer heat wave days has lowered productive performance by disturbing physiological homeostasis, reducing FI, and slowing growth rate [8,9]. Additionally, pigs exposed to HS exhibit thermoregulatory responses, including increased respiratory rate to enhance evaporative heat loss, reduced metabolic heat production due to decreased FI, and cutaneous vasodilation that increases peripheral blood flow to facilitate dry heat loss. Additionally, FI in growing-finishing pigs decreases by approximately 20% under HS, which likely reduces the heat increment of feeding [10,11]. In addition, HS drives a shift toward glucose centered metabolism and, through mitochondrial dysfunction and tissue hypoxia, increases reactive oxygen species (ROS) production [12,13]. The resulting ROS weaken tight junction (TJ) complexes, increase intestinal permeability, and facilitate the translocation of luminal endotoxin and pathogenic microbes, thereby triggering systemic inflammatory responses [14,15]. Systemic inflammation can dysregulate the hypothalamic pituitary gonadal (HPG) axis and impair gonadal steroidogenesis, thereby disrupting sex hormone homeostasis and reproductive performance [16]. Despite the growing body of HS research, translation across studies remains challenging because HS models and environmental reporting vary widely, key endpoints are often evaluated in isolation, and quantitative magnitudes relevant to production outcomes are not consistently consolidated [17–19]. To address these gaps, this review uses the temperature–humidity index (THI) to quantify heat load and harmonize heterogeneous HS conditions. We integrate evidence across endocrine and metabolic regulation, gut barrier function with the microbiota and immune response, and reproduction, and we summarize representative ranges of change in key parameters while linking mechanistic responses to productive and reproductive consequences.

## PHYSIOLOGICAL RESPONSES OF PIGS TO HEAT STRESS

In thermal contexts, HS occurs when environmental heat load exceeds an animal’s capacity for heat dissipation, leading to loss of thermal equilibrium [20]. Pigs have limited capacity for sweating, a relatively thick subcutaneous fat layer, and a low surface area to mass ratio, predisposing them to body heat storage and increasing their reliance on respiratory evaporative heat loss and cutaneous vasodilation for heat dissipation. Consequently, under comparable thermal conditions, pigs are more susceptible to HS than other livestock species [7]. To restore homeostasis under HS, the body activates the hypothalamic pituitary adrenal (HPA) and sympathetic adrenomedullary (SAM) axes, triggering coordinated endocrine, autonomic, and behavioral responses. With repeated or prolonged exposure, allostatic load accumulates and drives adverse reprogramming of FI, metabolism, immunity, intestinal barrier function, and reproductive endpoints [21,22].

The THI is the most widely used metric for quantifying HS; in this review, we adopt the formulation commonly applied in livestock practice [22].


(1) THI=([1.8×T]+32)-(0.55-0.55×[RH/100])×([1.8×T]+32-58)

Here, *T* denotes air temperature and *RH* denotes relative humidity. This index is an established metric for quantifying and standardizing heat load in livestock settings and is widely used to evaluate productive performance and physiological responses in pigs and to inform feeding and environmental management guidelines [23]. Reported threshold THI values derived using this equation depend on study design and the assumed relative humidity (RH). For sows, a commonly used four tier classification comprises ≤74, 74 to 78, 78 to 84, and >84 (Figure 2) [22,23]. These THI thresholds mark points at which pigs begin to experience HS and are associated with endocrine, physiological, and metabolic changes. Prolonged exposure to high ambient temperatures disrupts thermal homeostasis in pigs and induces a range of physiological abnormalities. Behavioral responses include reduced FI, peripheral vasodilation, and increased respiratory rate [8,18]. Across controlled studies summarized in Table 1, HS reduced FI by approximately 80% to 90%, increased respiratory rate by ~68% to 532%, and increased rectal temperature by ~1% to 6.6% relative to thermoneutral (TN) conditions (Table 1) [24–30]. Reduced FI is a major proximate driver of HS related growth depression in pigs. For example, finishing pigs exposed to temperatures at least 1.5°C above the baseline environmental temperature showed approximately 14% lower FI and 10% lower average daily gain (ADG) [31]. Consistent with this, a published meta-analysis of more than 80 trials reported that increasing ambient temperature reduces average daily FI (ADFI) and ADG in a curvilinear manner, with larger reductions in heavier pigs [32]. Representative controlled studies likewise report approximately 12% to 32% decreases in ADFI and 11% to 42% decreases in ADG under HS [9]. Changes in feed efficiency expressed as the gain:feed (G:F) are more variable because ADG and ADFI often decline concurrently, and TN pair feeding can also yield a low G:F, indicating that reduced intake may account for part of the apparent efficiency loss [29]. Importantly, intake controlled experiments also show differences in fat accretion between HS and TN pigs at the same FI, suggesting that HS can directly alter physiological and metabolic pathways beyond intake effects [9,33]. Together, these findings support the need for studies that disentangle intake mediated and direct physio metabolic effects of HS and clarify the underlying mechanisms.

## ENDOCRINE AND METABOLIC RESPONSES TO HEAT STRESS IN PIG

### Endocrine and metabolic mechanisms under the stress response and hypothalamic pituitary adrenal axis activation

Stress-induced endocrine and metabolic responses are coordinated physiological adjustments that occur when internal or external stressors activate the central nervous system, thereby engaging the SAM and HPA axes (Figure 3) [34]. Stress responses center on activation of the HPA axis. Stressors activate hypothalamic corticotropin releasing hormone (CRH) neurons, leading to CRH release. Corticotropin releasing hormone stimulates adrenocorticotropic hormone (ACTH) secretion from the anterior pituitary and concurrently recruits the SAM axis to mount physiological responses [35]. Secreted ACTH stimulates the adrenal cortex, promoting steroidogenesis, enhancing cholesterol uptake, and increasing the secretion of aldosterone and adrenal androgens [36]. In this process, absorbed cholesterol is converted to glucocorticoids (GC), thereby raising plasma GC concentrations, which in turn stimulate hepatic gluconeogenesis and enhance catecholamine stimulated lipolysis via β-adrenergic receptors [37]. In addition, energy redistribution to stress responsive tissues help maintain homeostasis and supports adaptation to stress [34,36]. However, prior studies report that cortisol, while promoting protein catabolism, also upregulates lipogenic enzyme expression, thereby driving the differentiation and maturation of preadipocytes [38]. This suggests that GC exert a dual function in stress conditions by coordinating both energy mobilization and storage.

### Endocrine crosstalk and tissue-specific metabolic responses under heat stress in pigs

Heat stress concurrently activates the HPA axis and the SAM axis, increasing cortisol and catecholamine secretion. These signals mediate the systemic stress response and promote energy mobilization through hepatic gluconeogenesis and adipose tissue lipolysis [37]. Moreover, unlike under TN conditions, pigs exposed to HS reduce FI yet maintain or even elevate circulating insulin; lipolysis in adipose tissue is relatively suppressed, and substrate utilization is reallocated toward hepatic and skeletal muscle pathways [13]. This response may be detrimental because reduced FI increases the need for lipid mobilization, yet suppressed adipose lipolysis constrains fatty acid availability. As a result, nutrient partitioning shifts toward glucose centered metabolism, which may compromise feed efficiency and growth under HS.

Insulin promotes energy storage by facilitating glucose uptake into skeletal muscle and adipose tissue and by enhancing lipogenesis and glycogen synthesis [39]. Rather than reflecting the action of a single hormone, metabolic homeostasis is maintained through coordinated crosstalk among cortisol, insulin, and catecholamines [35]. Although these endocrine responses are systemic, tissues differ in hormonal sensitivity and response profiles. Notably, skeletal muscle, a principal site of whole body protein turnover, exhibits increased insulin resistance under HS [40]. This is accompanied by increased expression of lactate dehydrogenase (LDH) in type II muscle fibers, establishing a metabolic milieu in which glycolysis predominates [41,42].

Consequently, protein and nucleic acid synthesis are suppressed, whereas proteolysis is accelerated, leading to increases in circulating catabolic markers such as blood urea nitrogen, 3-methylhistidine, and creatine phosphate [43]. These changes indicate that HS impedes intramuscular protein accretion and can be a major contributor to growth retardation.

### Adaptive reprogramming of energy metabolism and reduced metabolic flexibility under heat stress

Heat stress profoundly affects whole body energy balance and induces negative energy balance (NEB) through reduced FI and increased energy expenditure [44]. In general, pigs in a state of NEB prioritize energy conservation over tissue growth and rely on metabolites generated from fat and protein catabolism as major energy sources [39]. However, HS induced NEB exhibits metabolic features that differ from a typical energy deficit state. Notably, lipolysis is suppressed and plasma non-esterified fatty acids (NEFA) are reduced, indicating HS specific endocrine and metabolic regulation [13,43].

This suppression of lipid mobilization (lipolysis) is closely linked to elevated insulin levels, and in adipose tissue, insulin upregulates genes involved in lipid uptake and synthesis, including lipoprotein lipase (LPL), fatty acid synthase (FAS), and cytosine-cytosine-adenine-adenine-thymine/enhancer-binding protein α, thereby activating lipid storage pathways [44]. In addition, under HS, the expression of genes involved in fatty acid transport and synthesis, including adipocyte protein 2, cluster of differentiation 36 (CD36), and fatty acid transport protein 6 (FATP6), together with activation of the glyceroneogenesis pathway, which involves glycerol kinase and phosphoenolpyruvate carboxykinase 1, has been reported, thereby facilitating re-esterification of circulating NEFA (Table 2) [13,45–53]. Consistent with this insulin dominant adipose response, lipid storage pathways are upregulated, including PCK1 (+415.6%), LPL (+403.4%), and FAS (+162%), whereas CD36 (−72%) and CPT1A (−55%) are reduced, indicating constrained fatty acid transport and β-oxidation capacity (Table 2). Although HS reduces FI and can create an energy deficit consistent with NEB, adipose tissue often shows an insulin dominant program with lower circulating NEFA and reduced lipolytic responsiveness, limiting lipid availability for oxidation and metabolic flexibility [13,46]. This shift may reduce productive efficiency and, in females, impair reproduction by disrupting ovarian steroidogenesis and oocyte maturation, consistent with reports of ovarian and luteal dysfunction and poorer oocyte maturation and quality under HS [14,15].

This transition lowers the efficiency of ATP production from fatty acid oxidation and serves as a key mechanism that limits metabolic flexibility. Heat stress also induces adaptive remodeling of carbohydrate metabolism by increasing pyruvate dehydrogenase kinase 4 (PDK4), which phosphorylates and inhibits pyruvate dehydrogenase, thereby reducing the entry of acetyl coenzyme A derived from pyruvate into the tricarboxylic acid cycle and shifting glucose metabolism toward glycolysis [29,54]. In intake-controlled comparisons, ileal PDK4 mRNA abundance was greater in HS than in TN and pair-fed TN pigs [52], indicating a heat load-specific, carbohydrate-biased adaptation rather than an effect of reduced FI alone. Reduced DLD, IDH2, NDUFS2, and PGM2 expression under HS suggests impaired glucose oxidation, TCA cycling, and electron transport chain (ETC) function, lowering mitochondrial capacity and diverting glucose to lactate [46,55]. As a result, lactate/pyruvate and NADH/NAD^+^ ratios rise, favoring anaerobic ATP production over oxidative metabolism [6,25]. While lactate was once viewed mainly as a driver of acidification and inefficiency [6,42], recent transcriptomic data suggest tissue-specific recycling. For example, Huau et al. [48] reported increased hepatic LDH B and Cori cycle activation under HS, consistent with lactate recycling into glucose via gluconeogenesis to sustain energy availability. Collectively, these findings suggest compensatory metabolic reprogramming that helps maintain flexibility during heat adaptation.

## GUT HEALTH IN SWINE UNDER HEAT STRESS

### Intestinal barrier dysfunction and immune activation under heat stress

The gastrointestinal tract provides the largest mucosal surface in contact with the external environment and serves as a primary barrier and immune interface with commensal and pathogenic microorganisms [12,13]. The intestinal epithelium forms a multilayered barrier that permits selective nutrient absorption while blocking exogenous antigens, and is central to gut health [56]. It provides a first line of defense through a secreted mucus layer formed by the gel forming mucin 2 and an epithelial adherent layer composed of membrane bound mucins mucin 1, mucin 3, and mucin 17 [56]. In addition, the TJ complex, comprising claudins, occludin, MarvelD3, and tricellulin, plays a key role in maintaining intestinal epithelial barrier integrity by regulating paracellular permeability and sealing intercellular junctions [56].

Intestinal epithelial and Paneth cells secrete antimicrobial peptides and cytokines, chemokines to activate immune defenses, regulate inflammation, maintain microbiota balance, and limit pathogen invasion [57]. In pigs, HS redistributes blood flow from the splanchnic circulation to peripheral tissues, causing mesenteric ischemia and hypoxia [14].

Intestinal hypoxia stabilizes HIF-1α by inhibiting pyruvate dehydrogenase enzymes, thereby inducing hypoxia responsive gene expression [58]. Hypoxia followed by reoxygenation increases electron leak from the ETC and raises mitochondria derived ROS. Disruption of Ca^2+^ homeostasis induces endoplasmic reticulum stress, triggering the unfolded protein response and activating nuclear factor κB (NF-κB) signaling [59]. This induces the overexpression of proinflammatory cytokines and, consequently, serves as a major pathway that exacerbates inflammation and injury of the intestinal epithelium.

Alongside these intracellular signaling pathways, HS impairs the mechanical stability of the cytoskeleton. Disruption of Ca^2+^ homeostasis activates myosin light chain kinase, which phosphorylates myosin light chain and triggers contraction of the actomyosin complex [60]. This results in reorganization and structural weakening of the TJ complex, with reduced expression of claudin-1, occludin, and ZO-1, thereby increasing intestinal permeability [61]. Consequently, TJ gaps widen, compromising epithelial barrier integrity. Also, disrupted TJ and increased intestinal permeability permit translocation of luminal endotoxin into the circulation, activating inflammatory signaling via TLR4 (Figure 4) [47]. Heat stress also activates heat shock factor and upregulates heat shock protein (HSP), which are cytoprotective within cells, whereas some HSP bind TLR4 and amplify innate immune signaling [62]. Taken together, HS activates key stress signaling pathways that impair intestinal epithelial barrier function and hyperactivate immune responses, thereby precipitating the breakdown of intestinal homeostasis and driving systemic inflammation.

### Physiological and pathological responses of the intestine under heat stress

Under HS, multiple pathways disrupt porcine intestinal epithelial structure and function, weakening mucus defenses, increasing oxidative stress, permeability, and inflammatory activation [28,63]. Endoplasmic reticulum stress triggers the unfolded protein response, which impairs goblet cell secretory function and reduces mucin 2 production, thereby weakening the mucosal protective layer and compromising the first line of defense against pathogenic microorganisms [61]. Concurrently, the excessive generation of ROS under HS causes oxidative injury at the cellular level through mitochondrial DNA damage, lipid peroxidation, and protein modification [56].

These cellular injuries are accompanied by structural changes in the intestine. Under HS, maximum villus height decreased by approximately 43.2%, crypt depth by approximately 25.72%, and the villus:crypt ratio by approximately 37.4% (Table 3) [64–71]. These alterations impair nutrient absorption and overall gut function. Under HS, TJ disruption reduces transepithelial electrical resistance by approximately 50% and increases luminal endotoxin translocation into the circulation by >2 fold, promoting endotoxemia and TLR4/NF-κB driven proinflammatory cytokine release [55,68]. Heat stress is also accompanied by perturbations of the gut microbiota, characterized by depletion of beneficial commensals and expansion of pathogenic taxa, resulting in dysbiosis that disrupts epithelial and immune cell homeostasis and destabilizes the gut immune axis [30].

Heat shock protein (HSP) expression plays a dual role. At early stages, HSP support protein folding and provide cytoprotection. However, sustained or excessive HSP expression can overactivate immune responses through interactions with TLR4, thereby amplifying tissue injury and inflammation [64].

### Alterations of the porcine gut microbiota and its interplay with intestinal injury under heat stress

Heat stress impairs the selective permeability of the intestinal epithelium and reshapes the gut microbiota, and these processes are mutually reinforcing [72]. Under short term HS, consistent shifts in β-diversity are observed: the relative abundances of *Lactobacillus*, *Bifidobacterium*, and members of *Bacteroidetes* decrease, whereas opportunistic and inflammation associated taxa increase, including Proteobacteria (Campylobacterales; Desulfovibrionaceae), Veillonellaceae (*Megasphaera*), and *Clostridium* sensu stricto 1 [30,73,74].

Findings for α-diversity are mixed and depend on age category, exposure duration, and the metric used. In finishing pigs, Shannon index tends to decrease after 7 d of HS. In weaned piglets after 3 weeks of HS, observed richness, phylogenetic diversity, and Shannon increase. In late gestation sows, α-diversity does not appear to differ appreciably. Overall, the evidence indicates that, rather than a uniform reduction in total diversity, HS more consistently produces a compositional shift centered on β-diversity [63,70].

Under chronic HS, these patterns are amplified, and temperature exerts the strongest main effect even when diet and management are held constant (Table 4) [72–77]. In particular, cyclic HS is associated with a higher relative abundance of *Corynebacterium*, whereas TN conditions are enriched in taxa involved in fiber fermentation and short chain fatty acids (SCFA) production, including *Prevotella* and the *Eubacterium hallii* group, suggesting temperature dependent differences in the conversion of dietary fiber to SCFA [75]. These compositional shifts coincide with a reduction in SCFA, which, by compromising the energy supply to colonocytes, attenuates HIF-1α dependent defensive responses and reduces the expression of TJ proteins, mucin2, and antimicrobial peptides [78,79]. Increased intestinal permeability under HS is associated with higher serum diamine oxidase (DAO) and D-lactate, lower secretory IgA, and histologic lesions including mucosal injury, hemorrhage, lymphocytic infiltration, and fewer goblet cells. Pathway analyses show enrichment of NOD-like receptor, Th17/IL-17, JAK–STAT, and IBD-related signaling, consistent with a proinflammatory immune bias [79,80]. Fecal microbiota transplantation studies further support causality, as microbiota from HS pigs induced mucosal atrophy and inflammatory infiltration (with early goblet cell hyperplasia) in recipient mice [77]. In late gestation, HS also alters maternal fecal/vaginal microbiota and piglet meconium, suggesting disrupted maternal to offspring transfer and greater early environmental seeding [81].

Overall, β-diversity analyses consistently distinguish HS from TN, indicating that HS reproducibly reshapes gut microbial community composition even when α-diversity changes are inconsistent [63,70]. This compositional remodeling is biologically relevant because it often involves loss of SCFA producing and other beneficial taxa with expansion of opportunistic taxa, a shift that can reduce SCFA availability, weaken barrier defenses, promote inflammation, and ultimately depress FI and growth performance under HS [30,63, 75–77]. These gut and inflammatory disturbances associated with HS may also have reproductive implications, particularly in late gestation when maternal fecal and vaginal microbiota and piglet meconium are concurrently altered, suggesting disrupted microbial transfer from the dam to the offspring [76]. In summary, HS weakens the HIF dependent barrier maintenance system via a triad of changes: depletion of beneficial taxa, expansion of opportunists, and depletion of SCFA, and it initiates and perpetuates a vicious cycle of mucosal injury, inflammation, and dysbiosis [73]. This suggests collapse of the axis linking the gut microbiota and the immune system under HS, explaining productivity losses and increased disease susceptibility.

## EFFECTS OF HEAT STRESS ON THE SOW REPRODUCTIVE CYCLE

### Hypothalamic pituitary gonadal axis dysregulation and reproductive impairments under heat stress

Summer high-temperature conditions disrupt physiological homeostasis in pigs and impair both productive and reproductive performance [16]. Heat stress is recognized as a major environmental driver of reproductive disorders, including delayed estrus, reduced conception rates, and failures of ovulation and implantation [6]. Consistent with seasonal infertility, farm records indicate that pregnancy rate decreases by approximately 6% points and litter size declines by about 1.5 piglets per farrowing during summer heat load compared with spring [81]. These reproductive impairments are associated with HS-induced activation of the HPA axis, which can interfere with HPG axis regulation and contribute to reproductive endocrine dysfunction [16].

The HPG axis begins with hypothalamic secretion of gonadotropin-releasing hormone (GnRH), which stimulates pituitary release of luteinizing hormone (LH) and follicle-stimulating hormone (FSH) and thereby governs follicular growth and maturation, ovulation, implantation, and other key reproductive processes [16,81]. Under HS, activation of the HPA axis increases secretion of ACTH and GC. Although GC contribute to adaptive responses to physiological stress, they suppress hypothalamic kisspeptin neurons, reduce GnRH secretion, and suppress HPG axis function [81]. Consequently, LH and FSH secretion declines, delaying or arresting follicular development and ovulation and ultimately leading to reproductive impairments [6].

### Impaired luteal function and uteroplacental metabolic dysregulation under heat stress

Beyond impairing follicular development, HS has been reported to adversely affect postovulatory corpus luteum formation and maintenance [82]. In sows under HS, reduced LH secretion, altered luteal function, and decreased circulating progesterone concentrations have been reported, and HS has been closely associated with implantation failure and impaired maintenance of pregnancy [83].

Heat stress shortens luteal lifespan and impairs luteal function by reducing blood flow and increasing oxidative stress and signaling disruption, which lowers progesterone and weakens pregnancy maintenance [84]. Heat stress also disrupts uterine energy metabolism and glucose transport for implantation and early development; placental transporter genes such as GLUT3 are downregulated, creating intrauterine energy imbalance with potential long term fetal effects [85].

Prolactin (PRL) is a pleiotropic hormone involved in lactation, reproduction, and immune regulation [82]. Changes induced by HS in ovarian PRL receptor and JAK/STAT signaling have been observed in gilts, raising the possibility that disturbed PRL signaling may modulate ovarian function under HS [86]. However, further work is required to clarify the magnitude and consistency of this contribution relative to other endocrine pathways. Additionally, HS perturbs insulin signaling and induces hyperinsulinemia, which increases ovarian insulin receptor expression at the mRNA and protein levels and enhances receptor phosphorylation [87]. In parallel, expression of steroidogenic genes, including steroidogenic acute regulatory protein and aromatase, together with upregulation of sulfotransferase 1E1 involved in E2 metabolism, has been reported [87]. Along with increased insulin sensitivity, these changes reprogram steroidogenic pathways and remodel ovarian metabolic and endocrine functions. They also activate the downstream phosphoinositide 3-kinase–AKT pathway, increasing AKT1 and forkhead box O3 mRNA and protein expression and raising phosphorylated AKT levels [87].

Forkhead box O3 is a transcription factor that suppresses primordial follicle growth and promotes apoptosis. Under HS, increased forkhead box O3 expression can impede normal follicular progression and disrupt the balance between autophagy and apoptosis [88]. Persistent apoptosis, together with ineffective clearance of damaged cells and accumulation of abnormal cells, can lead to tissue dysfunction and reduced fertility [88].

### Long term physiological and performance changes in F1 piglets following prenatal heat stress

Heat stress alters fetal metabolic programming and induces long term effects on postnatal physiology and performance in offspring [89]. When maternal temperature exceeds the TN range, placental adaptation is impaired, reducing uteroplacental blood flow and nutrient delivery [90]. This dysfunction reduces amino acid and glucose transport and oxygen supply, delaying fetal development and contributing to intrauterine growth restriction (IUGR) [85,91].

During HS in sows, placental solute carrier family 2 member 3 (SLC2A3) mRNA decreases, SLC mediated nutrient transport pathways are broadly downregulated, and protein levels of glucose transporter 2 and peptide transporter 1 are reduced [85,91]. These changes indicate reduced transplacental nutrient transport and increased IUGR risk, with the proportion of piglets weighing ≤1.1 kg reaching 24.2% during HS, approximately 53% higher than under TN conditions [90,92].

These developmental impairments alter body composition, increasing fat deposition and suppressing muscle development. These changes are interpreted as a consequence of fetal metabolic reprogramming toward lipogenesis [92]. In skeletal muscle, dysregulated microRNA expression has been reported, leading to impaired myofiber formation and reduced expression of growth factors [93]. This lipid biased metabolic reprogramming and inhibition of myogenesis during fetal HS persist into postnatal life [92].

Piglets exposed to HS show higher thermoregulatory set points and greater maintenance energy requirements, resulting in lower protein accretion, higher fat deposition, and impaired feed efficiency [94]. Consequently, loin eye area declines and shear force rises, indicating reduced muscle mass and poorer meat quality [89,94].

At the molecular level, disturbances in skeletal muscle microRNA networks and RNA epigenetic marks have been reported, together with upregulation of lipogenic pathways including sterol regulatory element-binding protein 1c, FAS, and diacylglycerol O-acyltransferase 1 [92,95]. The gut immune axis also shows pronounced long term effects. In IUGR piglets from HS pregnancies, expression of TJ proteins such as ZO-1 and occludin is reduced, the nuclear factor erythroid 2-related factor 2/Kelch-like ECH-associated protein 1 antioxidant axis is weakened, and TLR4, NF-κB, and extracellular signal-regulated kinase signaling are activated. In parallel, SCFA decline and the microbiota is skewed, leading to a cascade of impaired barrier function, inflammatory increases in permeability, and heightened early life susceptibility to infection, with potential repercussions for later reproductive performance and across generations [72,96]. Across studies, prenatal exposure to HS in males is associated with reduced total sperm output, increased sperm morphological abnormalities, and delayed pubertal testicular development. In females, HS exposure is linked to diminished estrus expression and ovulatory function, lower fertilization and pregnancy maintenance rates, and a longer weaning to estrus interval, collectively reducing reproductive efficiency [89]. Moreover, maternal HS programs F1 physiology and metabolism, and these alterations may affect the F2 intrauterine environment and embryonic development, suggesting that productivity risks associated with HS can be transmitted across generations [89].

Consequently, HS affects postnatal piglets by disrupting physiological homeostasis, metabolic flexibility, immune competence, and reproductive tract development. These effects extend beyond fetal growth to reduced performance and later reproductive capacity, indicating persistent biological and economic impacts.

## MITIGATION STRATEGIES AND FUTURE RESEARCH DIRECTIONS FOR HEAT STRESS IN SWINE

Mitigating HS requires integrated strategies, with environmental control remaining the primary means of reducing heat load through precision climate management, including THI- or effective temperature-guided ventilation, zone cooling, and conductive cooling pads [97,98]. These measures are often implemented as hybrid systems that optimize thermal relief while considering humidity, air quality, and energy efficiency. However, HS-induced endocrine-metabolic dysregulation and gut barrier dysfunction may persist despite environmental interventions alone, underscoring the complementary role of nutritional strategies. Nutritional interventions generally aim to reduce metabolic heat increment [98], enhance antioxidant capacity, and preserve gut barrier function [16,18,99]. Under HS-associated reductions in FI and energy availability, diets with higher energy density and lower crude protein may improve nutrient utilization and maintain performance [17,99]. In addition, antioxidant and cytoprotective nutrients, including vitamin E, selenium, and betaine, may support redox homeostasis and attenuate inflammatory signaling [99], whereas probiotics and prebiotics may help alleviate dysbiosis and reinforce barrier integrity [100]. Future research should clarify mechanistic links among endocrine-metabolic reprogramming, gut barrier disruption, and microbiota remodeling through integrative multiomics approaches across reproductive stages [77]. Precision nutrition strategies integrating real-time physiological monitoring with individualized feeding warrant further evaluation under thermal stress, while longitudinal and multigenerational studies are needed to define the developmental and epigenetic consequences of gestational HS and to inform resilient breeding and management strategies.

## CONCLUSION

This review integrates THI based evidence and summarizes how HS, via activation of the HPA and SAM axes and crosstalk among insulin, cortisol, and catecholamines, reorients substrate use toward glucose while constraining lipid mobilization. This metabolic shift involves PDK4 mediated inhibition of pyruvate dehydrogenase together with downregulation of TCA cycle and ETC activity. At the organ level, hypoxia, along with oxidative and endoplasmic reticulum stress, weakens TJ and mucin 2, increases paracellular permeability, and, in concert with dysbiosis, SCFA depletion, and TLR4/NF-κB hyperactivation, entrenches a self-perpetuating disruption of the axis linking the gut microbiota and the immune system. The magnitude and direction of these effects vary with covariates such as growth stage, thermal environment, breed, parity, and body weight, making prior covariate standardization essential for both research reporting and field application. To address the limitations of single metrics, this review adopts an integrative multi omics and microbiome perspective to map mechanistic links, highlighting that a PDK4 centered bias toward glucose metabolism and a downshift in TCA and ETC activity align with SCFA loss, reductions in TJ/mucin 2, and TLR4/NF-κB hyperactivation to drive gut immune dysregulation. Nevertheless, compared with physiological and production endpoints, multi omics integration and causal verification of HS associated microbiome changes in pigs remain limited. Future studies should combine standardized HS models with causal frameworks such as pair feeding and fecal microbiota transplantation to evaluate and refine the decision support value of microbiome, metabolic, barrier, and inflammation panels. From a commercial production standpoint, HS increases performance variability by depressing intake, impairing gut health, and compromising reproductive efficiency, thereby elevating economic risk during summer heat events. Accordingly, THI based heat load characterization and covariate aware interpretation provide practical value for benchmarking herd risk and for evaluating integrated mitigation packages under conditions.

## Figures and Tables

**Figure 1:**
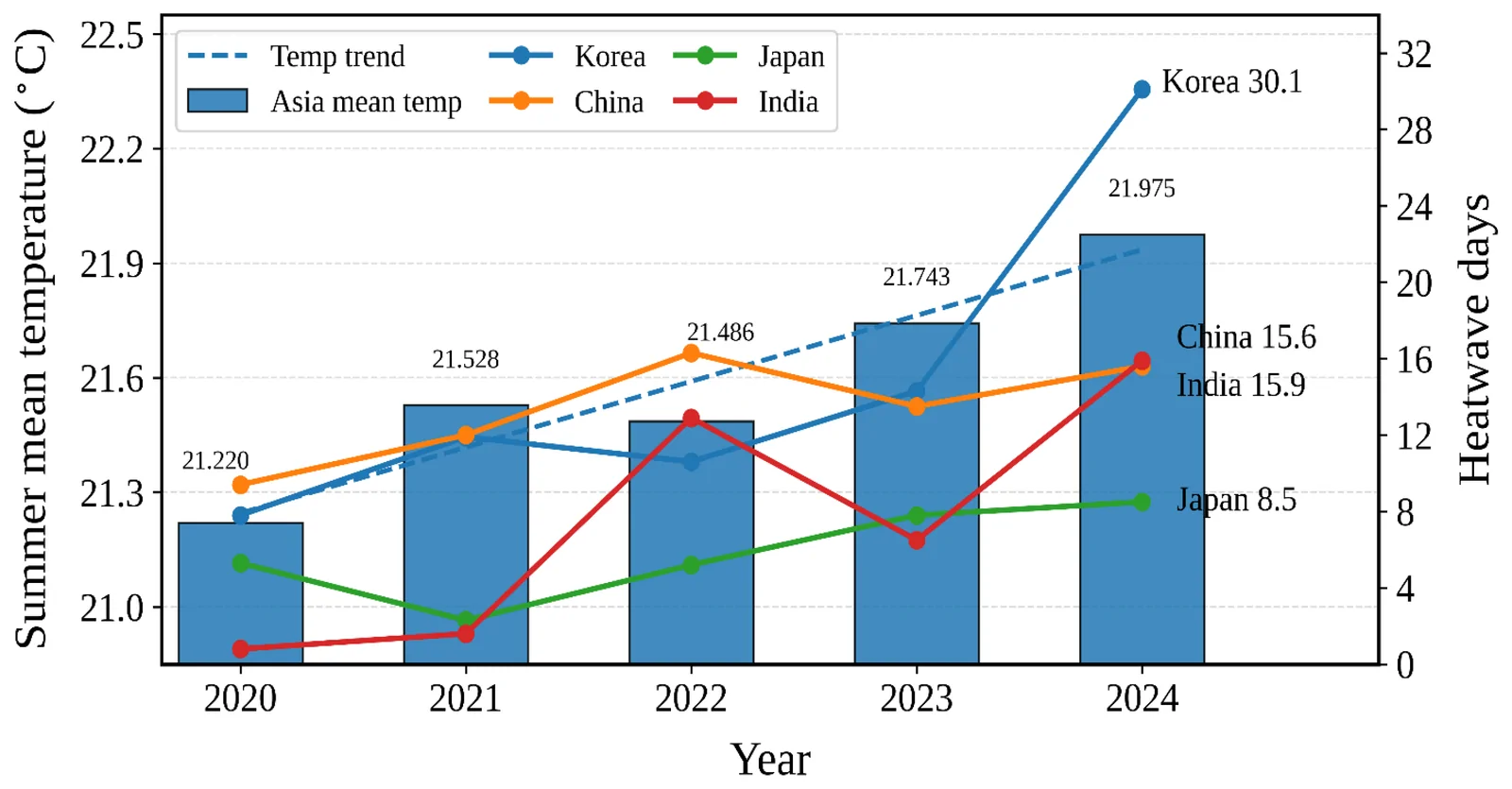
Asia June to August mean temperature and annual heat-wave days in four Asian countries (2020 to 2024). The bars depict Asia’s June to August mean air temperature (°C), and the lines depict annual heat-wave days for Korea (≥33°C), Japan (≥35°C; 13-station mean), China (≥35°C; national average), and India (IMD subdivision average = total).

**Figure 2:**
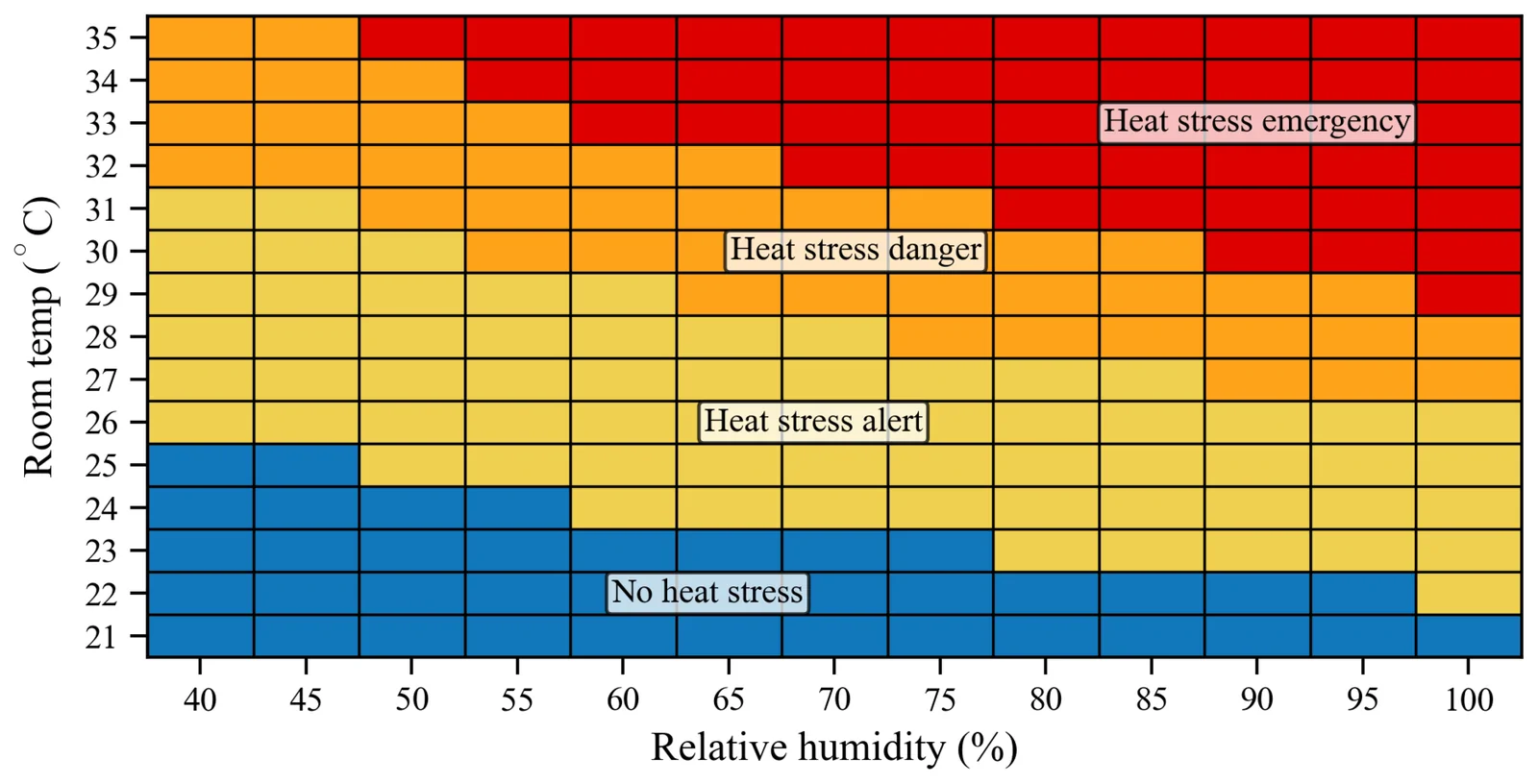
temperature–humidity index (THI) reference table for estimating heat stress in pigs. Ambient temperature and relative humidity are shown. Risk is classified into four levels: no stress (THI<74), alert (74 to 78), danger (78 to 84), and emergency (THI>84). Temperature humidity index was calculated according to the NOAA [22] equation.

**Figure 3:**
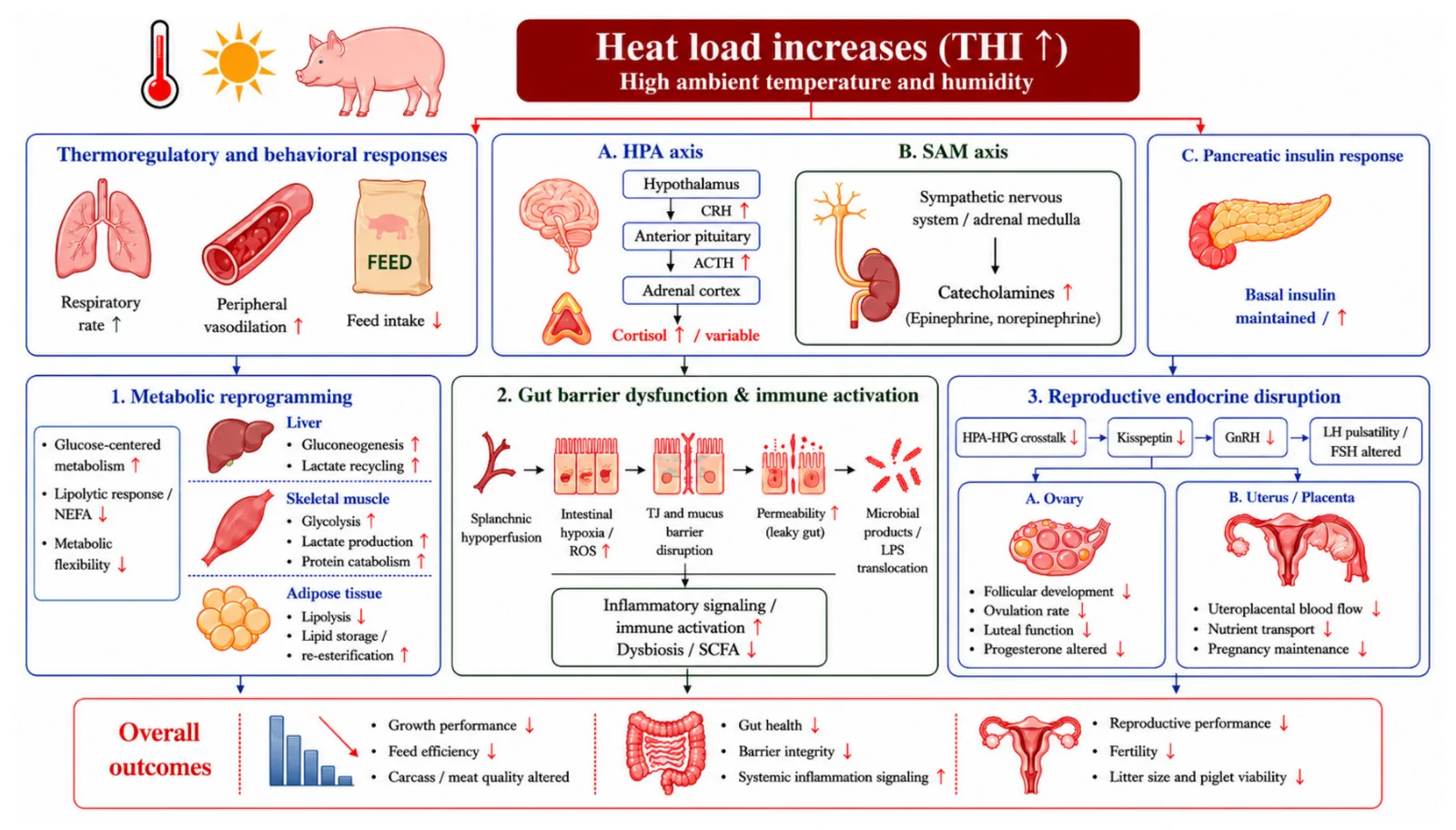
Integrated physiological consequences of heat stress in swine. Increased heat load, expressed as elevated temperature–humidity index (THI), induces thermoregulatory and behavioral responses, including increased respiratory rate, peripheral vasodilation, and reduced feed intake. Heat stress also activates neuroendocrine responses through the hypothalamic–pituitary–adrenal (HPA) axis, sympathetic–adrenomedullary (SAM) axis, and pancreatic insulin-related pathway. These responses promote glucose-centered metabolic reprogramming, hepatic gluconeogenesis and lactate recycling, skeletal muscle glycolysis and protein catabolism, and reduced adipose lipolytic response/non-esterified fatty acids (NEFA) availability. In parallel, heat stress impairs intestinal barrier function and immune homeostasis and disrupts reproductive endocrine regulation, ultimately reducing growth performance, gut health, fertility, litter size, and piglet viability. ↑, increase; ↓, decrease. LPS, lipopolysaccharide; SCFA, short chain fatty acids; LH, luteinizing hormone; FSH, follicle-stimulating hormone.

**Figure 4:**
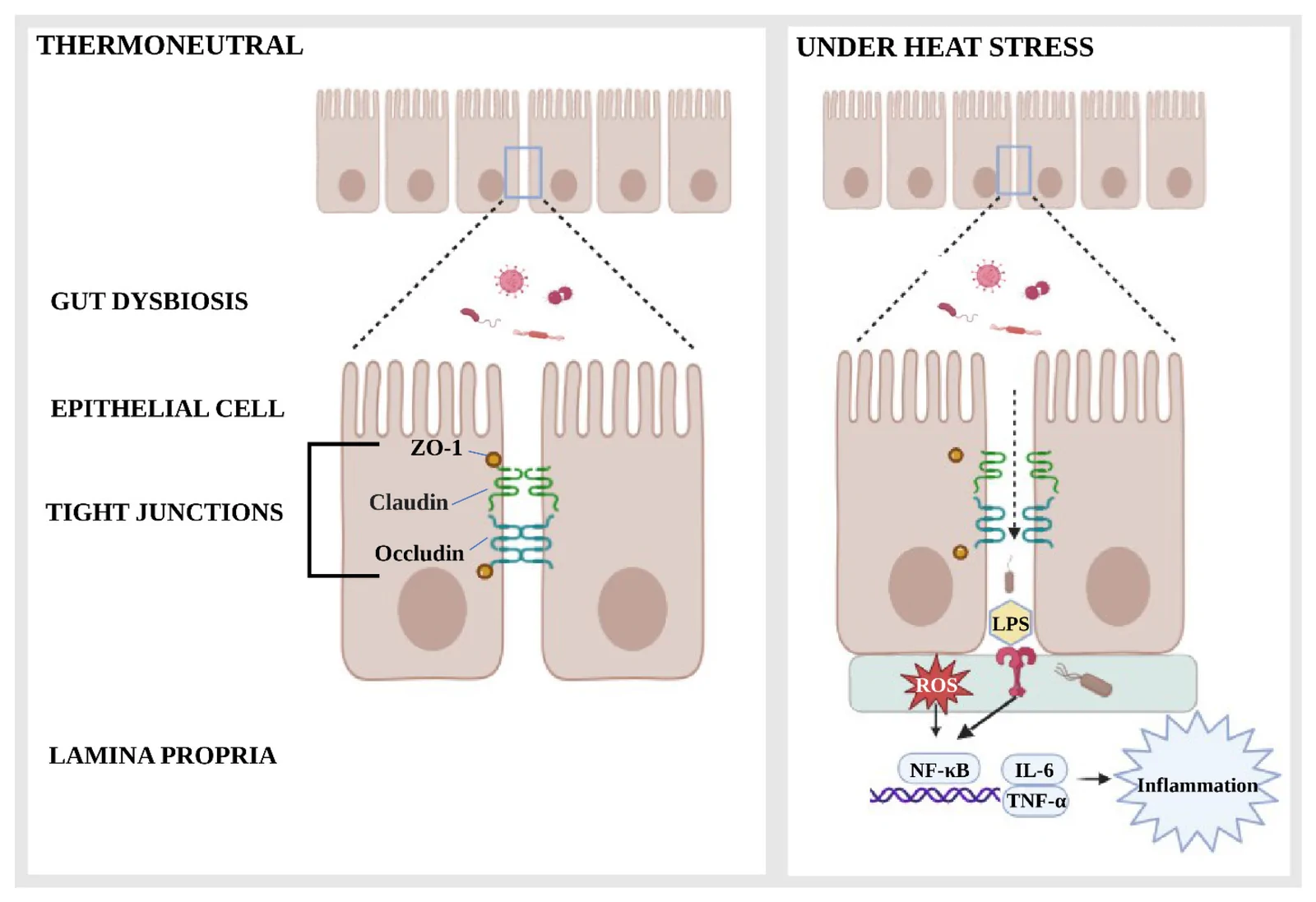
Disruption of the intestinal epithelial barrier and inflammatory signaling by heat stress via LPS, TLR4, and NF-kB. Compared with TN conditions (left), heat stress (right) disrupts TJ complexes (ZO-1, claudins, occludin) and increases paracellular permeability, allowing luminal LPS to translocate. Lipopolysaccharide (LPS), TLR4 signaling, together with ROS, activates NF-κB, upregulating pro-inflammatory cytokines that enter the circulation and drive systemic inflammation. ROS, reactive oxygen species; NF-κB, nuclear factor κB; IL-6, interleukin-6; TNF-α, tumor necrosis factor-α; TJ, tight junction.

**Table 1:** Percent changes in feed intake, respiration rate, and rectal temperature of swine under heat stress

Reference	Environmental conditions	THI	Heat stress model1)	Parameters	Change (%)2)
[24]	TN: 21.9±0.5°C, 62±13% RH, 24 h/ HS: 29.7±1.3°C, 49±8% RH, 24 h	TN: 66.8 HS: 78.1	Constant/acute	FI	−31.8**
				RR	76.3**
				RT	2.62**
[30]	TN: 25±1.0°C, RH 75% to 85% HS: 35±1.0°C, RH 75% to 85% 24 h	TN: 68.12 HS: 77.7	Constant/acute	FI	−53.8**
				RR	180.6**
				RT	5.9%**
[29]	TN: 23±3.0°C, RH: 68±10% HS: 33°C, RH: 58±10% (08:00 to 18:00 h) HS: 28, RH: 56±8% (18:00 to 08:00h) 21 d	TN: 67.6 HS: 80.7 (08:00 to 18:00 h) HS: 74.6 (18:00 to 08:00 h)	Cyclic/chronic	FI	−28.6**
				RR	156.9**
				RT	2.1**
[25]	TN: 22.0±0.4°C, RH 43% to 63%, 5 d HS: 33.6±0.5°C RH 22% to 40%, 5 d	TN: 67.6 HS: 86.6	Constant/short-term	FI	−7.7
				RR	67.9**
				RT	1.0
[26]	TN: 19°C, RH: 61% 7 d HS: 36°C, RH: 50% 7 d	TN: 64 HS: 85.5	Constant/short-term	FI	−59**
				RR	213.9**
				RT	1.3**
[27]	TN: 20°C, RH 35% HS: 35°C, RH 35% (09:00 to 17:00 h) 2 d HS: 28°C, RH 35% (17:00 to 09:00 h) 2 d	TN: 65.8 HS: 86.9 HS: 77	Cyclic/acute	FI	NR
				RR	532**
				RT	3.4**
[28]	TN: 21°C (RH: 70%) 12 h HS: 28°C (RH: 40%) 12 h	TN: 65.3 HS: 89.7	Constant/acute	FI	−89.6**
				RR	266.5**
				RT	6.63**
[18]	TN: 20°C RH 35% to 45% HS: 35°C, RH 35% to 45% (09:00 to 17:00 h) 8 d HS: 28°C, RH 35% to 45% (17:00 to 09:00 h) 8 d	TN: 65.8 HS: 86.9 (09:00 to 17:00 h) HS: 77 (17:00 to 09:00 h)	Cyclic/chronic	FI	−36.8**
				RR	352.9**
				RT	4.12**

1) Acute: ≤2 d, short-term: 3 to 7 d, chronic: >7 d; environmental conditions and THI describe the thermal exposure (TN vs. HS). The reported “change (%)” values refer to changes in response parameters under HS relative to TN, not to changes in temperature or THI, calculated according to NOAA [22].

2) p<0.01 = **; Change (%) = ([HS−TN]/TN)×100; % percentage change under HS relative to TN.

THI, temperature–humidity index; TN, thermoneutral; RH, relative humidity; HS, heat stress; FI, feed intake; RR, respiration rate; RT, rectal temperature; NR, not reported.

**Table 2:** Effects of heat stress on porcine metabolic pathways

Pathway	Reference	Environmental conditions	THI1)	Heat stress model2)	Marker	Effect3)
Lipid metabolism						
	[46]	HS: 35±1°C, 45% to 65% RH, 7 d	83.8	Constant/short-term	*PCK1*	3.3 vs 0.64 (415.6%)**
	[46]	HS: 35±1°C, 45% to 65% RH, 7 d	83.8	Constant/short-term	*LPL*	4.38 vs 0.87 (403.4%)**
	[50]	HS: 35°C 30 d	NR	Constant/chronic	*CD36*	0.28 (−72%)*
	[13]	HS: 32°C, 23% RH, 8 d	76.2	Constant/short-term	*FASN*	0.44 vs 0.17 (162%)**
	[53]	HS: 35°C, RH: 70, 10 d	79.6	Constant/chronic	*CPT1A*	0.45 vs 1.00 (−55.0%)*
Glucose metabolism						
	[48]	HS: 35°C 5 d	NR	Constant/short-term	*DLD*	0.73 (−26.8%)*
	[49]	HS: 28°C to 34°C 61.1% RH	77.79	Cyclic/chronic	LDH	2,141 vs 2,288 (−6.4%)*
	[51]	HS: 33°C, 60% RH, 24 h	80.38	Constant/acute	*IDH2*	0.6 (−40.3%)**
	[51]	HS: 33°C, 60% RH, 24 h	80.38	Constant/acute	*NDUFS2*	0.38 (−61.6%)**
	[47]	HS: 30°C, 55% RH, 21 d	77.53	Constant/chronic	*PGM2*	1.92 (−48%)*

1) Calculated according to NOAA [22].

2) Acute: ≤2 d, short-term: 3 to 7 d, chronic: >7 d.

3) p<0.05 = *, p<0.01 = **; Change (%) = ([HS−TN]/TN)×100 When only fold change was reported results are shown as “FC (HS/TN) (Change, %)”. Percent change was calculated as ([HS−TN]/TN)×100 for the “value vs value” format and as (FC−1)×100 for the FC format.

THI, temperature–humidity index; HS, heat stress; RH, relative humidity; NR, not reported; *PCK1*, phosphoenolpyruvate carboxykinase 1; *LPL*, lipoprotein lipase; *CD36*, cluster of differentiation 36; *FASN*, fatty acid synthase; *CPT1A*, carnitine palmitoyl transferase; *DLD*, dihydrolipoamide dehydrogenase; LDH, lactate dehydrogenase; *IDH2*, isocitrate dehydrogenase 2 (NADP^+^), mitochondrial; *NDUFS2*, NADH ubiquinone oxidoreductase core subunit S2; *PGM2*, phosphoglucomutase 2.

**Table 3:** Effects of heat stress on intestinal morphology

Reference	Environmental conditions	THI1)	Heat stress model2)	Parameters	Change (%)3)
[68]	TN: 20±1°C, RH: 35% to 50% HS: 35±1°C, RH: 20% to 35%	TN: 65.7 HS: 89.4	Constant/short-term	Villus height	Jejunum: −20.53*
				Crypt depth	Jejunum: −4.50*
				V:C ratio	Jejunum: −17.65*
[66]	TN: 23.8±0.1°C, RH: 41.1% HS: 33.6°C, RH: 25% (19:01 to 06:59 h) HS: 38.3°C, RH: 25% (07:00 to 19:00 h)	TN: 70.8 HS: 87.7 (19:01 to 06:59 h) HS: 95 (07:00 to 19:00 h)	Cyclic/short-term	Villus height	Jejunum: −16.90** Ileum: −11.30**
				Crypt depth	Jejunum: −10.90** Ileum: 3.40
				V:C ratio	Jejunum: −6.90 Ileum: −13.90*
[71]	TN: 23°C, RH: 60% HS: 40°C, RH: 60% (04:00 to 09:00 h) 26°C RH: 60% (09:00 to 04:00 h)	TN: 68.3 HS: 88.8 (04:00 to 09:00 h) HS: 71.9 (09:00 to 04:00 h)	Cyclic/chronic	Villus height	Duodenum: −9.63* Jejunum: −10.45* Ileum: −1.66
				Crypt depth	Duodenum: −4.92 Jejunum: −12.55 Ileum: −13.41
[65]	TN: 21.2±0.31°C, RH: 61.9±6.9% (6 h) HS: 38.5±0.60°C, RH: 36.4±3.4% (HS: 3 h, TN: 3 h)	TN: 66 HS: 92.7	Constant/acute	Villus height	Jejunum: −24.72* Ileum: −26.11*
				Crypt depth	Jejunum: −2.50 Ileum: −1.26
				V:C ratio	Jejunum: −24.03* Ileum: −25.29*
[69]	TN: 21°C, RH: 70% HS: 37°C, RH: 40%	TN: 65.3 HS: 89.7	Constant/acute	Villus height	Ileum: −36.88*
				Crypt depth	Ileum: −4.67
				V:C ratio	Ileum: −34.69*
[70]	TN: 25±1°C, RH: 75% to 85% HS: 35±1°C, RH: 75% to 85%	TN: 68.6 HS: 78.7	Constant/chronic	Villus height	Duodenum: −33.94* Jejunum: −37.44* Ileum: −43.20*
				Crypt depth	Duodenum: −11.14 Jejunum: −25.24* Ileum: −9.03*
				V:C ratio	Duodenum: −26.32* Jejunum: −13.87 Ileum: −37.40*
[67]	TN: 20°C HS: 28°C (20:00 to 06:00 h) 35°C (06:00 to 20:00 h) 28 d	NR	Cyclic/chronic	Villus height	Jejunum: −5.65 Ileum: −9.71
				Crypt depth	Jejunum: −7.89 Ileum: −6.71
				V:C ratio	Jejunum: 3.85 Ileum: −3.57
[64]	TN: 24.2±0.5°C, RH: 57.2±5.6% HS: 33.6±1.8°C, RH: 29.5±5.3% (18:00 to 07:00 h) HS: 37.4±2.1°C, RH: 29.5±5.3% (07:00 to 18:00 h) 3 d	TN: 70 HS: 86.9 (18:00 to 07:00 h) HS: 92.6 (07:00 to 18:00 h)	Cyclic/short-term	Villus height	Jejunum: −16.93* Ileum: −11.26*
				Crypt depth	Jejunum: −10.94** Ileum: 2.00*
				V:C ratio	Jejunum: −6.87* Ileum: −13.91*
				Villus width	Jejunum: 6.78* Ileum: −15.51*

1) Calculated according to NOAA [22].

2) Acute: ≤2 d, short-term: 3–7 d, chronic: >7 d.

3) p<0.05 = *, p<0.01 = **; Change (%) = ([HS–TN]/TN)×100.

THI, temperature–humidity index; TN, thermoneutral; RH, relative humidity; HS, heat stress; V:C ratio, villus height-to-crypt depth ratio; NR, not reported.

**Table 4:** Effects of heat stress on intestinal microbiota composition

Reference	Animal/phase	Sample	Thermal model	α-Diversity	β-Diversity	Phylum-level differences1)	Genus-level differences
[77]	Growing	Ileum & cecum mucosa	TN 23±1°C HS 33±1°C, 72 h	NR	PERMANOVA (Bray–Curtis): HS, TN R^2^ = 0.14; p<0.05	NR	*Lawsonia* ↑, *Actinobacillus* ↑, *Chlamydia* ↑; *Flavonifractor* ↓, *Bifidobacterium* ↓·*Staphylococcus* ↑, *Akkermansia* ↑; *Lactobacillus* ↓, *Prevotella* ↓, *Succinivibrio* ↓
[30]	Growing-finishing	Fecal	TN 25±1°C HS 35±1°C, 24 h	Simpson ↓ (p<0.05)	ANOSIM (Bray-Curtis) R = 0.28; p<0.01	Proteobacteria ↑, Bacteroidetes ↓ (p<0.05)	NR
[63]	Primiparity sows	Fecal	TN 18 to 22°C HS 28 to 32°C, 30 d	NR	NMDS (Bray–Curtis) ANOSIM p = 0.016	NR	Ruminococcaceae UCG-005 ↑, [*Eubacterium*] coprostanoligenes group ↑, Ruminococcaceae UCG-013 ↑, *Coprococcus* 3 ↑, *Halomonas*; ↓ *Streptococcus* ↓, Bacteroidales RF16 group (norank) ↓ (p<0.05)
[74]	Growing-finishing	Fecal	Acute HS 29.0±1.3°C Chronic HS NR	Acute HS: richness (observed OTU) ↑ (p<0.05). Chronic HS: Shannon p<0.05)	NR	Acute HS Firmicutes ↑, Bacteroidetes ↓, Spirochaetes ↑, Fibrobacteres ↑, Tenericutes ↑ Chronic HS Firmicutes ↑, Proteobacteria ↑, Spirochaetes ↑; Bacteroidetes ↓, Actinobacteria ↓ (Kruskal–Wallis, p<0.05)	Prevotellaceae-affiliated OTU ↑; Lachnospiraceae-affiliated OTU ↑; Clostridiaceae 1-affiliated OTU ↓; Peptostreptococcaceae-affiliated OTU ↓ (edgeR GLM, FDR<0.05)
[72]	Growing pigs	Ileum & cecum digesta	TN 23°C HS 33°C, 21 d RH 55%±5%	Cecum richness (Chao, phylum-level) ↑, p = 0.020	NR	Cecum Bacteroidota ↓, p = 0.011	*Mycoplasma* ↑ *norank_Erysipelotrichaceae* ↑ *Aeriscardovia* ↓ *Clostridium* sensu stricto_1 ↑ *Coprococcus* ↓
[75]	Finishing	Fecal	CHS: 22°C (20:01 to 07:59 h), 35°C (08:00 to 20:00 h); TN: 22°C	NR	PERMANOVA (Bray–Curtis): day 27 R^2^ = 0.09; p = 0.004; all samples R^2^ = 0.03; p = 0.006 · ANOSIM (unweighted UniFrac): day 27 R = 0.11; p = 0.008	NR	*Corynebacterium* ↑
[76]	Primiparity sows, neonatal piglets	Sow: fecal and vaginal swab, Piglet: fecal	TN 18°C to 22°C HS 28°C to 32°C	NR	PERMANOVA (Bray–Curtis): sow feces p<0.01, vagina p<0.01, piglet meconium p<0.01	Sow feces: Spirochaetes ↓ · Vagina: Bacteroidetes ↑, Fusobacteria ↑ · Piglet: Proteobacteria ↑, Firmicutes ↓	Sow feces HS ↑: Ruminococcaceae UCG-005, [Eubacterium] coprostanoligenes group HS ↓: Roseburia Vagina HS ↑: Bacteroides, Actinobacillus HS ↓: Anaerococcus Piglet (meconium) HS ↑: Acinetobacter, Klebsiella

1) Phylum- and genus-level differences indicate taxa whose relative abundance was significantly altered under heat stress compared with TN conditions; ↑, increased under heat stress; ↓, decreased under heat stress. Only significant differences are shown unless otherwise indicated.

TN, thermoneutral; HS, heat stress; NR, not reported; PERMANOVA, permutational multivariate analysis of variance; ANOSIM, analysis of similarities; NMDS, non-metric multidimensional scaling; OTU, operational taxonomic unit; GLM, generalized linear model; FDR, false discovery rate; RH, relative humidity; CHS, cyclic heat stress.

## Data Availability

Upon reasonable request, the datasets of this study can be available from the corresponding author.
